# Protura in Arctic Regions, with Description of *Mastodonentomon* n. gen. (Acerentomidae, Nipponentominae) and a Key to Known Arctic Taxa

**DOI:** 10.3390/insects11030173

**Published:** 2020-03-09

**Authors:** Julia Shrubovych, Jerzy Smykla, Ernest C. Bernard

**Affiliations:** 1 Institute of Systematics and Evolution of Animals, Polish Academy of Sciences, Sławkowska 17, 31-016 Kraków, Poland; shrubovych@gmail.com; 2 State Museum of Natural History, Ukrainian National Academy of Sciences, Teatral’na St. 18, UA 79008 L’viv, Ukraine; 3 Institute of Soil Biology, Biology Centre Czech Academy of Sciences, Na Sádkách 7, 370 05 České Budějovice, Czechia; 4 Institute of Nature Conservation, Polish Academy of Sciences, Mickiewicza 33, 31-120 Kraków, Poland; 5 University of Tennessee, Entomology and Plant Pathology, 2505 E.J. Chapman Drive, 370 Plant Biotechnology, Knoxville, TN 37996, USA; ebernard@utk.edu

**Keywords:** proturans, arthropods, chaetotaxy, porotaxy, distribution, biogeography, Alaska, Siberia, Northern Canada, Beringia

## Abstract

Protura are widespread, but their presence in the Arctic was first noted only ca. 70 years ago and is still little acknowledged. This work compiles taxonomic information on proturans in the Arctic regions and adds unpublished data from Northern Siberia. Currently, this fauna is represented by 23 species in two orders and 14 genera. The large cosmopolitan genus *Eosentomon* is represented by only four species, whereas Acerentomidae is much more diverse, with 19 species in 13 genera (eight Nipponentominae, five Acerentominae). Most of the Arctic species possess a larger number of setae than species living in temperate regions. Based on several unique characters, a new genus, *Mastodonentomon*, is erected for *Nipponentomon macleani*, and the species is re-described with the original description supplemented with new characters, including head chaetotaxy, seta length, and porotaxy. Proturan occurrence in the Arctic is limited to Beringia, but the majority of species have restricted distributions and none have been found in both the American Arctic and Siberia. This implies relict origins and high levels of proturan endemism in the Arctic. This emerging view on biogeographical history is, however, hampered by the limited extent of available data, which highlights the need for considerably greater survey efforts. A key to Arctic proturans is provided to facilitate further studies.

## 1. Introduction

Protura, known as coneheads, are among the smallest of soil-dwelling microarthropods. These hexapods are wingless, eyeless, and have slender elongate bodies ranging between 0.7–2 mm. Due to their minuteness and cryptic edaphic lifestyle, they are easily overlooked and were discovered relatively late in the history of entomology [1]. Originally regarded as an order belonging to the class Insecta [2], Protura was later placed among the basal hexapod taxa as the sister taxon to either Collembola or Diplura. Their phylogenetic position, however, is still a point of controversy [1,3]. Since their first description in 1907 [2], over 800 valid species belonging to three orders, seven families, and 76 genera have been described worldwide [1,4,5]. However, this number likely represents only ca. 10% of the world’s species [6].

Proturans usually live alongside other microarthropods, such as Acari, Collembola, Pauropoda and Symphyla, with which they form an essential component of edaphic communities in all terrestrial regions and climatic zones, and elevations ranging from sea level to the treeless zone on high mountains [1,7]. Their distribution seems to be limited only by the presence of sufficient moisture levels and the availability of decaying organic matter [7,8]. Although proturans are sometimes common, they are infrequently collected, usually only as a by-catch of other arthropods. Thus, their diversity and geographical and ecological distributions are still little known [1,7].

Some authors have also pointed to the general lack of proturans in polar regions and nival zones of high mountains [1,4,9]. While proturans have never been found in the Antarctic and Sub-Antarctic regions, they are recorded above the Arctic Circle in tundra and taiga biomes, reaching past 72° N. However, only a few papers concern the study of proturan fauna in the Arctic regions. For instance, until now, only Tuxen [10], Nosek [11,12,13], and Bernard [14] have documented the occurrence of Protura in Alaska and the Canadian Arctic. Nosek [13] also summarized the data on proturan fauna and presented a key for the identification of all proturan species known at that time from Alaska. Data about Arctic Protura from the Palearctic region also are very scarce [15,16,17,18]. Recent advances in proturan taxonomy highlight the need for taxonomic revisions, and several species from Alaska and Northern Canada have been re-described and transferred to other genera [19,20,21,22].

In this work, all data on proturans from the Arctic regions available from the literature are reviewed and summarized in order to get a clearer idea of the true diversity and distribution of these arthropods. Proturans from soil and moss samples collected recently in Northern Yakutia (Siberia) were also extracted and analysed, which significantly extends the known northernmost limits of their distribution. Furthermore, an integrative re-description of *Nipponentomon macleani* Nosek, 1977 is provided and a new genus, *Mastodonentomon*, is established for this species. A key for determination of all proturans known from the Arctic regions is also provided to facilitate further studies.

## 2. History of Proturan Research in the Arctic

Protura were first collected inside the Arctic Circle by M. Hammer, who, in 1948, collected well over 100 specimens from two different locations above 68° N in Northern Canada [10]. The majority of these proturans were described as *Acerentulus canadensis* Tuxen, 1955, and six specimens from one sample as *Nosekiella condei* Tuxen, 1955 (Table 1). Currently, these species are in the genera *Verrucoentomon* and *Vesiculentomon*, respectively [19,22]. Hammer’s collection contained one specimen belonging to the genus *Eosentomon*. It was, however, a “larva 2” and, therefore, indeterminable to species [10]. Weber [23] mentioned a record of “*Acerentulus* sp. (Family Acerentomidae)” collected in 1949 from two locations in Alaska, extending the northernmost proturan record past 69° N. Then it was not until summer 1976 when a notable collection of proturans from a number of localities across Alaska was made by S.F. MacLean and A. Fjellberg (Institute of Arctic Biology, Fairbanks AK) [11] with the northernmost localities above 70° N. Based on that collection, Nosek [11,12,13] described 14 species, including three Eosentomidae and 11 Acerentomidae, and wrote a key [13] for determining all proturan species known from Alaska. Lastly, Bernard (1985) [14] described *Eosentomon adakense* from samples collected in 1978 on Adak Island (Aleutian Islands, Alaska). Some of the Acerentomidae species have been transferred to other genera: *Orinentomon*, *Sugaentulus*, and *Imadateiella* [19,20,24]. The genus *Nosekientomon* was erected for the species *Vesiculentomon ruseki* Nosek, 1977 [19]. Two *Alaskaentomon* species and *Verrucoentomon imadatei* Nosek, 1977 are valid and were re-described recently [21,22]. Revision of the type material of *Nipponentomon macleani* Nosek, 1977 shows that this species has some unique characters within Acerentomidae and a new genus has to be established for it.

The first known Arctic proturan in the Palearctic was collected from Snow Valley in the Magadan district in Siberia: *Acerella sharovi* Martynova, 1977 [15]. This species was transferred to *Imadateiella* by Imadaté [25]. Three proturan species were described from the taiga belt near Turukhansk City, close to the Arctic Circle [16,18,26], and *Nienna chukotka* Shrubovych, 2019, was described from Northern Chukotka above 69° N [27]. This record and a new record of *Yamatentomon yamato* Imadaté & Yosii, 1956, from northern Yakutia above 72° N, are currently the northernmost known Palearctic localities of Protura.

## 3. Materials and Methods

### 3.1. Faunistic Dataset

A dataset of the distribution of proturans in Arctic regions was created based on the publications concerning these species. In addition, new data from Northern Yakutia (Siberia) reported in the current work were also included. Geographically, the dataset covers all studies conducted in the Arctic, as defined by the Arctic Monitoring and Assessment Programme (AMAP) [28], including all the terrestrial areas north of the Arctic Circle (66°33′ N) as well as most areas north of 60° N in Asia and North America with continuous permafrost, and the Aleutian Islands. It should be borne in mind that a significant proportion of these areas are often defined as Subarctic, but we follow the AMAP terminology for simplicity.

The dataset (Table 1) includes: (1) the valid species names and synonyms, and their taxonomic position, (2) sites where proturans were collected and their coordinates (with the type locality marked with *), (3) type of habitat in which they were found, as given in the original descriptions, (4) remarks on their known distribution, and (5) literature sources of the original records and species re-descriptions if available.

### 3.2. Material Examined

Proturan specimens from Northern Yakutia (Siberia) were extracted from soil and moss samples with Berlese-Tullgren funnels into 95% ethanol. The specimens were mounted on glass slides in Faure’s medium [29] for taxonomic evaluation. The examined specimens were deposited in the collection of the Institute of Systematics and Evolution of Animals, Polish Academy of Sciences, Kraków (ISEA). In addition, the type material (holotype female and paratype male) of *Nipponentomon macleani*, preserved in the National Museum of Natural History (Smithsonian Institution) in Washington, DC, USA, was also studied using oil immersion and phase contrast on an Olympus microscope.

The terminology for body chaetotaxy and porotaxy follows Szeptycki [30] and Shrubovych [18]. The head seta designations follow Rusek et al. [31]. Abbreviations used in the description are as follows: Abd. = abdominal segments, Th. = thoracic segments, *A*-setae = anterior setae, *M*-setae = medial setae, *P*-setae = posterior setae, *al* = anterolateral, *sl* = sublateral, *psm* = posterosubmedial, *psl* = posterosublateral, *spm* = sternal posteromedial, and *spsm* = sternal posterosubmedial cuticular pores.

## 4. Results

### 4.1. Faunistic Dataset

**Figure 1:**
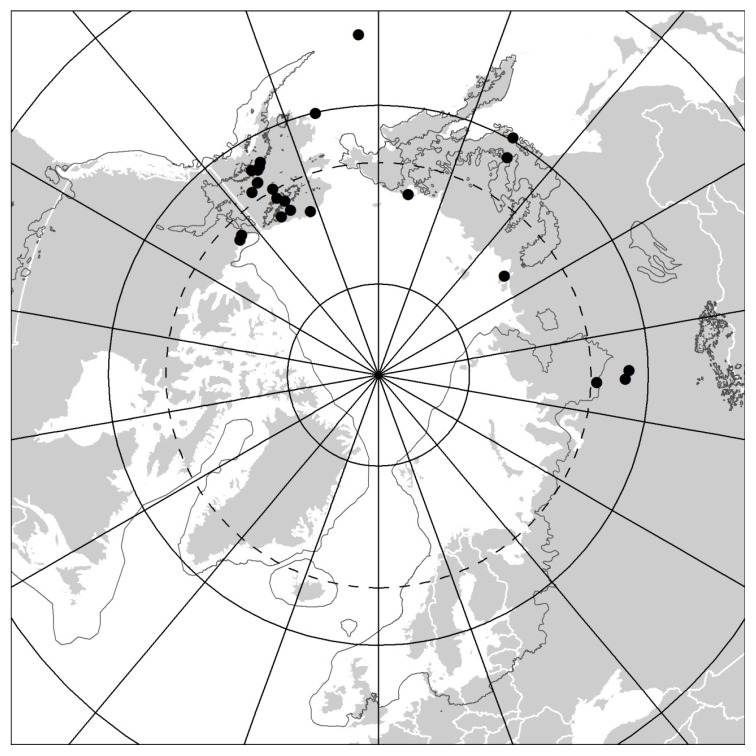
Map showing all known sites of Protura occurrence within the Arctic regions. The dark grey irregular line shows the largest ice-sheet extent during the Pleistocene Last Glacial Cycle (redrawn after Reference [32]).

The faunistic dataset contains 37 records of Protura from 24 sites within the Arctic regions (Table 1), published originally in 11 papers. Of these, 26 records from 17 sites and 6 papers [10,11,12,13,14,23] concern Alaska and the Canadian Arctic and only 11 records from 7 sites and 5 papers [15,16,17,26,27] concern the Siberian Arctic. No records were found from the European Arctic (Figure 1). Overall, 23 proturan species from two orders (Eosentomata and Acerentomata) and 14 genera were identified from both the North American and Siberian Arctic (17 and 6 species, respectively). The order Eosentomata is represented by only four species, which all belong to the genus *Eosentomon*. Acerentomata are considerably more diverse, with 19 species belonging to 13 genera (eight Nipponentominae and five Acerentominae). Moreover, two records undetermined to species level, both reported as “*Acerentulus* sp. (Family Acerentomidae)” [23], were listed from Northern Canada. Unfortunately, Weber [23] did not provide any descriptions and whereabouts of these collections are unknown, so they could not be re-examined. It should also be remembered that, since that time, *Acerentulus* has been split into several genera. Thus, at present, these records can be assigned, at best, to Acerentomidae only.

Species richness for particular sites ranged from one to four even though the majority of sites (18) hosted only single species and only three sites (i.e., Chena Ridge and Denali National Park in Alaska, and Turukhansk in Siberia) had four species. However, it should be borne in mind that these estimates are mostly based on historical data taken from the literature and they might be biased by the sampling effort. Nearly all species reported from Alaska and the Canadian Arctic (i.e., 13 of 17 species) are known only from their type localities and only four were also found in other sites across this region. On the other hand, among Siberian species, only *Nienna chukotka* Shrubovych, 2019 is known exclusively from its type locality. The other five species seem to have broader distributions across Siberia and records of *Yamatentomon yamato* (Imadaté and Yosii, 1956), reported in the present paper from the northernmost locality, span a wide latitudinal range across the Northeastern Palearctic. It is also remarkable that none of the species have been found in both regions.

### 4.2. New Record from the Arctic Region

*Yamatentomon yamato* (Imadaté & Yosii, 1956).

**Material Examined:** 2 females, 9 males, 1 maturus junior, 4 larvae II, 7 larvae I, soil and moss in tundra. Russia, Sakha-Yakutia, Tyallakh, Lena Delta Nature Reserve, sedge/moss tundra near the reserve station, 72°19′36″ N, 128°07′27″ E, 16.08. 2015., coll. A. Nekhaeva.

**Distribution:** Japan, Korea, Northeastern China, Russian Far East [4,18] and Northern Siberia (current study).

**Remarks:** The specimens are identical with more southern specimens (see References [18,33]) in body length, chaetotaxy, and porotaxy. The sensilla pattern on the foretarsus is similar, with the very long sensillum *b* surpassing the seta *γ4* insertion point. Unlike southern specimens, foretarsal sensillum *a* is shorter. Its apex reaches to the sensillum *t2* insertion, and does not reach the base of sensillum *d*. This small character difference is not enough to justify a description of a new species.

### 4.3. Systematics

#### 4.3.1. ***Mastodonentomon*** Shrubovych, New Genus

Characterized by the presence of four pairs of *A*-setae on meso- and metanota *(A1*, *A2*, *A3*, *A4*), setae *M2* and *M3* on metanotum, six pairs of *A*-setae on tergites I–III (additional setae *A1’* present), five pairs of *A*-setae on tergites IV–VII, filiform foretarsal sensillum *t1*, sensillum *b’* missing, position of foretarsal sensillum *b* proximal to *c* insertion, *d* situated closer to *e* than to *c*, sensillum *a’* in distal position, close to *c’* insertion, well-developed maxillary, and labial palps with terminal tuft of setae, posterior position of *P3* setae on tergites II–VI, well-developed striate band on segment VIII with distinct parallel-sided striae, 4/2 setae on sternite VIII, and presence of two setae of equal length on abdominal legs II and III.

**Type species:***Mastodonentomon macleani* (Nosek, 1977).

**Remarks:** The new genus *Mastodonentomon* is placed into subfamily Nipponentominae due to possessing a distinct calyx with large vesicle and racemose appendices on its surface, presence of four pairs of *A*-setae on metanotum, presence of 4/2 setae on sternite VIII, well-developed striate band on segment VIII, and two setae of equal length on abdominal legs II and III [5]. The new genus is similar to *Nipponentomon* and *Imadateiella* in having four pairs of *A*-setae on metanotum and filiform foretarsal sensillum *t1*. The genus differs in the presence of five pairs of *A*-setae on tergite VII and in the position of foretarsal sensillum *a’*, which is placed far distally, close to *c’* insertion, as in some *Acerentomon* spp. [34,35]. The positions of sensillum *a’* on the foretarsus clearly separates most of the genera within Nipponentominae [19]. This new genus differs from all other Acerentomidae in the presence of four pairs of *A*-setae on the mesonotum (*A1*, *A2*, *A3*, *A4*) and three pairs of *M*-setae (*M1*, *M2*, *M3*) on the metanotum. Other Protura have only two or three pairs of *A*-setae on mesonotum and one pair of *M*-setae on the metanotum. These characters are important at the generic level and justify the erection of *Mastodonentomon* n. gen. within the subfamily Nipponentominae.

#### 4.3.2. ***Mastodonentomon macleani*** (Nosek, 1977), New Combination

**Figure 2:**
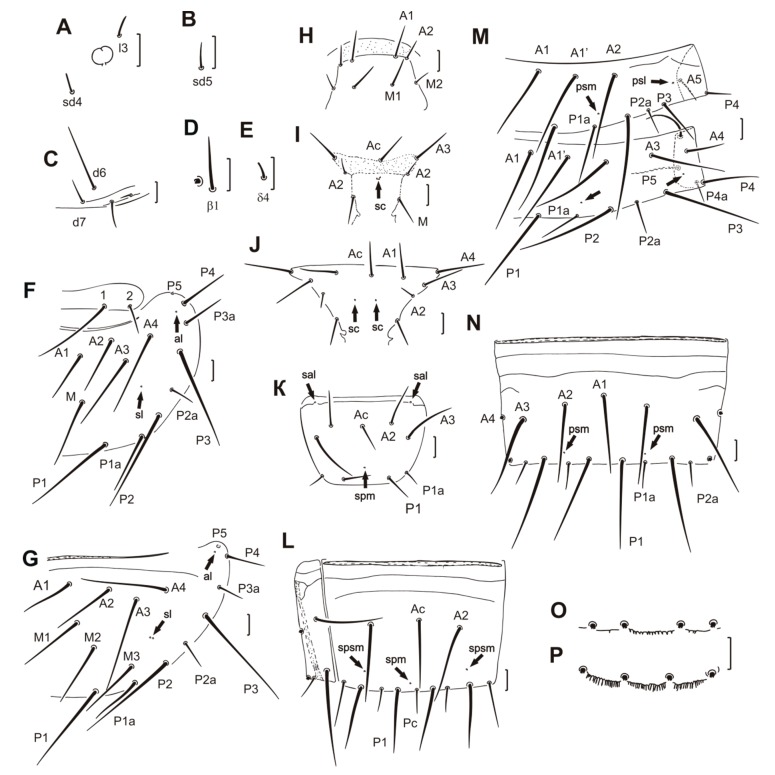
*Mastodonentomon macleani* (Nosek, 1977). (**A**) Pseudoculus with setae *l3* and *sd4*. (**B**) Cephalic seta *sd 5.* (**C**) Hind part of head. (**D**) Foretarsal seta *β1*. (**E**) Foretarsal seta *γ4*. (**F**) Pronotum and mesonotum, right side. (**G**) Metanotum, right side. (**H**) Anterior part of the prosternum. (**I**) Anterior part of the mesosternum. (**J**) Anterior part of the metasternum. (**K**) Sternite I. (**L**) Sternite VII. (**M**) Tergites I and II, right side. (**N**) Tergite VII. (**O**) Hind margin of tergite IX. (**P**) Hind margin of tergite X. Arrows indicate pores (sl = sublateral, psm = posterosubmedial, psl = posterosublateral, sc = sternal central, spm = sternal posteromedial, spsm = sternal posterosubmedial). Figures A–E, J: paratype. Figures F–I, K–P: holotype. Scale bars = 20 µm.

(Figure 2A–P, Table 2)

syn. *Nipponentomon macleani* Nosek, 1977

**Material Examined:** Holotype female (no. 75801), collected in *Dryas* on exposed cliff, Mastodon Dome at Eagle Creek, 1200 m elev., Alaska, 1.VIII.1976, coll. A. Fjellberg. Paratype male collected together with the holotype. The slides are mounted in Swan’s medium and stored in the National Museum of Natural History (Smithsonian Institution) in Washington, USA.

**Diagnosis:** Large species with a body length of more than 1500 μm. Setae *A1* present on mesonotum, metanotum, and metasternum, setae *M2* and *M3* on metanotum, six pairs of *A*-setae on tergites I–III, five pairs of *A*-setae on tergite VII, five *A*-setae on sternites I–VI and three *A*-setae on sternite VII. Additional cephalic seta *d6* very long; sensilla *f* and *c’* the longest on foretarsus. Pores *al* and *sl* present on meso- and metanota, group of *sc* pores present on mesosternum, two separate *sc* pores present on metasternum, *psl* present on tergites I–VII, pore *spm* present on sternites I–VII, a pair of *sal* pores present on sternite I, and a pair of *spsm* pores on sternites VI–VII.

**Re-description:** The maxillary and labial palpi, canal of the maxillary gland, arrangement of the foretarsal sensilla, setae of the third abdominal legs, striate band and comb on abdominal segment VIII, female squama genitalis were all accurately depicted by Nosek [11] to which the reader is referred for illustrations.

Head setae *l3*, *sd4*, and *sd 5* setiform, 15, 13, and 16 μm in length, respectively (Figure 2A,B). Additional setae *d6* very long, 65–70 μm (Figure 2C). Labrum not protruded. LR = 16. Pseudoculus round with a short posterior extension. PR = 21. Calyx of maxillary gland with two large racemose appendices. Posterior filament long, with simple posterior dilation. CF = 5.0–5.2 (see Reference [11]: Figure 6E). Maxillary palp with terminal tuft of setae. Sensilla short and pointed apically, similar in length and shape (see Reference [11]: Figure 6C). Labial palps with long, broad basal sensillum (see Reference [11]: Figure 6C).

On foretarsus, sensillum *t1* filiform, *t3* small and leaf-like. *f* and *c’* very long, nearly setiform (see Reference [11]: Figure 6A,B). Sensillum *b* shorter than *c* and situated proximally to level of *c* insertion, *g* short and slender. Sensillum *d* closer to *e* than to *c*, *a’* in distal position, close to *c’* insertion, *b’* missing. Length formula of sensilla: *t3* < *t1* < (*b* = *g*) < (*a* = *d*) < (*t2* = *c* = *e* = *a’*) < *c’* < *f*. Seta *β1* setiform and long, seta *δ4* short and stout, their length 32 μm and 10 μm, respectively (Figure 2D,E). Claw long, with long inner tooth, empodial appendage long. BS = 0.6–0.8, TR = 2.4–2.6, EU = 0.14–0.15. Single pores near bases of sensilla *c* and *t3*.

Chaetotaxy formula given in Table 2. Setae on nota differing in length (Figure 2F). Pronotal seta *1* three times longer than seta *2*. Meso- and metanota with seta *A1*. Seta *P1a* on meso- and metanota long, setiform, seta *P2a* short, half length of seta *P1a*; *P2a* situated midway between *P2* and *P3*. Length ratio of mesonotal setae *P1*:*P1a*:*P2* as 1.6:1:1.8. Mesonotum with a pair of single *sl* and *al* pores and metanotum with a pair of doubled *sl* pores and a pair of *al* pores (Figure 2F,G). Prosternum without pores, mesosternum with a group of three closely placed *sc* pores, metasternum with a pair of separately placed *sc* pores (Figure 2H–J). Seta *A2* on thoracic sterna and *P4* on tergite I short, setiform (Figure 2I,J,M).

Tergites I–III with additional *A1’* setae (Figure 2M). All accessory setae on tergites I–VI setiform. Setae *P1a* longest on tergite I (60 μm), shorter on tergites II–VI (about 43 μm), and shortest on tergite VII (37 μm). Setae *P2a* shorter than *P1a*, their length on tergite I 27 μm, on tergites II–VI 34 μm. Setae *P4a* the shortest when compared to other accessory setae, their length 20 μm on tergite II and 25 μm on tergites III–VII. Setae *P2a* and *P4a* on tergite VII 25 μm long. Pores *psm* and *psl* present on tergites I–VII anterior to *P1a*, *al* on tergites II–VII (Figure 2M,N).

Abdominal legs with 4, 2, 2 setae. Subapical and lateral apical setae on second and third pairs of abdominal legs nearly equal in length, 25 and 23 μm, respectively (see Reference [11]: Figure 6F). Lengths of accessory setae *P1a* on sternites I–III 17, 20, and 23 μm, respectively, of equal length (26 μm) on sternites IV–VII. Sternites I–V with single *spm* pores, sternite I with a pair of *sal* pores (Figure 2K). Sternites VI–VII with *spm* pores and a pair of *spsm* pores anterior to *P2* (Figure 2L).

Abdominal segment VIII with a distinct striate band. Tergite and sternite each with anterior, irregular, transverse row of small teeth (see Reference [11]: Figure 7). Pore *psm* with several accompanying teeth. Posterior margin of sternite VIII and laterotergites smooth. Comb VIII with 9–10 long teeth (see Reference [11]: Figure 6G). Sternites IX–X with central setae half the length of lateral setae (see Reference [11]: Figure 6H). Tergite IX with minute serrations in the central part of the hind margin (Figure 2O), tergites X–XI, and sternite X with distinct serrations on the entire posterior margin (Figure 2P). Sternite IX with serrations on the lateral part of the hind margin, sternite X with serrations on the entire posterior margin (see Reference [11]: Figure 6H). Dorsal lobe of tergite XII with a median pore and a ventral lobe with 1+1 anterolateral pores.

Female squama genitalis with long, apically bipartite acrostyli (see Reference [11]: Figure 6I). Male squama genitalis with 6 + 6 setae.

**Measurements:** 2 adults (in μm): total length 1570–1660, head 235, pseudoculus 11, posterior part of maxillary gland 45–47, posterior cephalic setae: seta *d6* 65–70, *d7* 21–22, seta *sd7* 25–26, seta *l5* 9, pronotal seta *1* 84–80, pronotal seta *2* 27; mesonotal seta *P1* 92, mesonotal seta *P1a* 54–56, mesonotal seta *P2* 103, foretarsus 150–151, claw 58–60, empodial appendage 10–12.

**Chaetal variability:** In the holotype, seta *A1* doubled on metasternum, *Pc* doubled on sternite III, *A1* absent asymmetrically on tergite VII. In paratype, seta *A1’* present symmetrically on tergite IV.

**Etymology:** The generic name is taken from the general locality (Mastodon Dome) where the specimens were collected.

**Remarks:** In this re-description of *Nipponentomon macleani*, morphological characters are added to the previous description of the species, such as chaetotaxy of head, length of seta on head, nota and foretarsus, and porotaxy on tergites and sternites. According to Nosek [11] tergite I has 6 *A*-setae and 14 *P*-setae. However, re-examination of the type material clearly showed that tergite I has 8 *A*-setae (seta *A5* present) and 12 *P*-setae.

### 4.4. Key to Arctic Protura

1.Meso- and metanotum with a pair of spiracles  ………………(*Eosentomon* Berlese, 1908) … 20– Meso-metanotum without spiracles  …………(Acerentomidae Silvestri, 1907) … 22.Metanotum with two pairs of *A*-setae ………………………… 3– Metanotum with three or four pairs of *A*-setae ……… 83.Abdominal legs II and III with two setae ……………………………………… 4– Abdominal legs II and III with three setae ………… *Fjellbergella tuxeni* Nosek, 19804.Striate band on tergite VIII well developedwith distinct striae ………………………… 5– Striate band reduced to two lineswithout striae …………… *Tuxenentulus boedvarssoni* Nosek, 19815.Foretarsal sensillum *t1* filiform ………………(*Alaskaentomon* Nosek, 1977) … 6– Foretarsal sensillum *t1* claviform  ……………(*Sugaentulus* Imadaté, 1978) … 76.Sternites II–III with five *A*-setaetergite VII without seta *P3a*
………… *Alaskaentomon condei* Nosek, 1981– Sternites II–III with three *A*-setaetergite VII with seta *P3a*
……… *Alaskaentomon fjellbergi* Nosek, 19777.Tergites I–VI with seta *P1a*, sternites IV and VI with four *A*-setae  …………………*Sugaentulus hoogstraali* (Nosek, 1980)– Tergites I–VI without seta *P1a*, sternites IV and VI with three *A*-setae  …………………*Sugaentulus andrzeji* Shrubovych & Rusek, 20108.Metanotum with three pairs of *A*-setae ………………………… 9– Metanotum with four pairs of *A*-setae ………………………… 159.Foretarsal sensillum *t1* filiform ………………………… 10– Foretarsal sensillum *t1* claviform ………………………… 1310.Calyx of a maxillary gland with small granulated appendicesforetarsal sensillum *t3* globular ………………………… *Nienna chukotka* Shrubovych, 2019– Calyx of a maxillary gland with large granulated appendicesforetarsal sensillum *t3* leaf-like ………………………… (*Verrucoentomon* Rusek, 1974) … 1111.Sternite I without setae *P1a*, sternite VII with seta *Pc*
………………………… 12– Sternite I with setae *P1a*, sternite VII without seta *Pc*
……… *Verrucoentomon anatoli* Shrubovych & Bernard, 201212.Foretarsal sensilla *b* and *c* equal in length ……… *Verrucoentomon canadense* (Tuxen, 1955)– Foretarsal sensillum *b* clearly longer than *c*
……… *Verrucoentomon imadatei* Nosek, 197713.Maxillary gland with smooth globular vesicle near calyx ……… *Nosekientomon ruseki* (Nosek, 1977)– Maxillary gland without vesicle near calyx  ………(*Yavanna* Szeptycki, 1988) … 1414.Foretarsal sensillum a longits apex reaching the base of seta *γ3* sensillum *g* thin  ……… *Yavanna behanae* (Nosek, 1977)– Foretarsal sensillum a shorterits apex not reaching base of seta *γ3* sensillum *g* thickened  ……… *Yavanna babenkoi* ShrubovychRusek & Bernard, 201215.Foretarsal sensillum *t1* filiform  ……… 16– Foretarsal sensillum *t1* baculiform or claviform  ……… 1816.Foretarsal sensillum *a’* in distal positioninsertion close to base of sensillum *c’*
 ……… *Mastodonentomon macleani* (Nosek, 1977)– Foretarsal sensillum *a’* in proximal positioninsertion near base of sensillum *t2*
 ……… (*Imadateiella* Rusek, 1974) … 1717.Tergite VII with three pairs of *A*-setae, sternite VI with *Pc* setasternite VII with six setae  ……… *Imadateiella sharovi* (Martynova, 1977)– Tergite VII with two pairs of *A*-setae, sternite VI without *Pc* setasternite VII with four setae  ……… *Imadateiella mixta* (Nosek, 1981)18.Foretarsal sensillum *t1* baculiform  ……… 19– Foretarsal sensillum *t1* claviform  ……… *Orinentomon greenbergi* (Nosek, 1980)19.Sternite VII with six setaesternite VI without *Pc* seta  ……… *Vesiculentomon condei* (Tuxen, 1955)– Sternite VII with four setaesternite VI with *Pc* seta  ……… *Yamatentomon yamato* Imadaté & Yosii, 195620.Foretarsal sensillum *b’1* presentsternite VIII without *A*-setae  ……… 21– Foretarsal sensillum *b’1* absentsternite VIII with a pair of *A*-setae  ……… *Eosentomon aquilinum* Nosek, 198021.Tergite VIII with three pairs of *A*-setae  ……… 22– Tergite VIII with two pairs of *A*-setae  ……… *Eosentomon adakense* Bernard, 198522.Foretarsal sensilla *t2* and *b’2* spatulate  ……… *Eosentomon copelandi* Nosek, 1980– Foretarsal sensilla *t2* and *b’2* setiform  ……… *Eosentomon alaskaense* Nosek, 1977

## 5. Discussion

The proturan fauna of the Arctic regions is not rich but is quite diverse, represented by two orders (Eosentomata and Acerentomata), 14 genera and 23 species (Table 1). The order Sinentomata has not been recorded from this region, but only five species are known at present [4]. Considering over 800 species of Protura known worldwide [1,4,18], the proportion of the global proturan diversity found in the Arctic is very low (less than 3%). Species richness recorded for particular sites was also very low and the majority of sites hosted only single species. Nevertheless, nearly 20% of 76 genera known worldwide [5] have been found in the region. Members of the widespread genus *Eosentomon*, which are very abundant and diverse in the Americas and Europe [36,37,38], are poorly represented, with only four known species, and restricted in distribution to Alaska and Northern Canada (Table 1). However, the lack of *Eosentomon* in the Siberian Arctic is not surprising because species of this genus are generally rare and not numerous in neighboring regions [18,39]. Acerentomidae is very diverse in the Arctic at the generic level and comprise eight genera of Nipponentominae and five of Acerentominae. The genus *Verrucoentomon* is most diverse in the Arctic fauna. Its members have usually been collected in mountainous regions, such as *V. montanum* (Martynova, 1970) and *V. rafalskii* Szeptycki, 1997. Such apparently relict species may have been able to survive on non-glaciated mountaintops [18,40]. The subfamilies Berberentulinae and Acerellinae apparently are absent from the Arctic regions. Berberentulinae is rich in species and widely distributed. Acerellinae is restricted to four species know only from Europe [4]. Members of these subfamilies are characterized by a reduction of some morphological characters, e.g., shape of labial palps, striate band, number of setae on body segments, and sensilliform accessory setae on the body. The acerentomids on the northern edge of proturan distribution possess well-developed labial palps with a terminal tuft of setae, well-developed striate band on segment VIII (except *Fjellbergella* and *Tuxenentulus* spp.), and a larger number of setae on the body than species with a more southern distribution.

In general, Protura can be found wherever decaying organic matter and sufficient moisture levels are available [1,8], and often are restricted to habitats characterized by luxuriant vegetation growth [7]. It is believed that proturans are fungivorous and some species may feed preferentially on ectomycorrhizal fungi [41]. It is not surprising, therefore, that proturans have rarely been collected in tundra biome, which marks the northern limit of their distribution. They have never been collected in the taiga biome in the European part of Russia, but proturans occur in Siberian taiga and tundra biotopes [42].

Alaska, Yukon, and Siberia are a part of the Beringian region that was mostly ice-free during the Pleistocene glaciations [32] and, therefore, proturans are found further north there than in the rest of the Holarctic. This suggests the importance of historical biogeography in the distribution of proturan species, which was a hypothesis highlighted by Tuxen [43]. Furthermore, records suggest pronounced biogeographical differences in distribution of proturan species within the Beringian region. For instance, no species were found in both the North American and Siberian Arctic. This disparity implies the existence of a strong intercontinental disjunction in proturan distributions between these two Arctic regions. Moreover, the majority of species seem to have regionally restricted distributions or are known only from their type localities. This pattern is particularly pronounced for the proturan fauna in North American Arctic, but also among Siberian species. Only *Yamatentomon yamato* is widely distributed over a broad latitudinal range extending far beyond the Arctic region (see Reference [18]). While substantial northward migration and post-glacial recolonization from the south is apparent from datasets for some previously glaciated regions, e.g., Southern Canada [44] or Southern Fennoscandia [4], it appears that further north in the Arctic regions distribution of proturans is still limited to the areas of Beringia that were unglaciated during the Pleistocene (Figure 1). Moreover, the restricted distributions and morphological differences among some taxa, when compared to species with a more southern distribution (as indicated above), imply a relict origin and a potentially very high level of endemism in proturan fauna of that region. This hypothesis is consistent with distribution patterns reported for some other arthropod taxa, particularly those with limited dispersal capabilities (see Reference [45]), which were presumably eliminated or prevented from dispersing by glaciers elsewhere, but for which Beringia acted as a refugium. However, the available data on proturan occurrence are fragmentary and restricted to very few specific collection localities. Many areas have not been sampled at all. Thus, the apparent restricted distributions may, in part, reflect the limited sampling intensity and location of specific collection sites rather than the real distribution of these animals. While our work provides both a benchmark for the region and the foundation for future research, considerably greater survey efforts are necessary to elucidate the true diversity of proturans and biogeographical patterns of their distribution in the Arctic regions.

## 6. Conclusions

Although proturans are often believed to be absent in polar regions, their occurrence above the Arctic Circle was noted more than 70 years ago with the northernmost records exceeding 72° N. While early works focused on documenting and describing new species, primarily in Alaska and the Canadian Arctic, recent advances in proturan taxonomy have facilitated re-descriptions of several taxa and documented the occurrence of proturans throughout Northern Siberia. Accordingly, in the current work, we re-describe *Nipponentomon macleani*, supplementing the original description with new characters, including head chaetotaxy, seta lengths, and porotaxy, and, on the basis of several unique characters, establish a new genus, *Mastodonentomon*, for this species. Nonetheless, the Arctic literature dataset contains only 35 records representing 23 species, i.e., less than 3% of the global proturan diversity. In contrast, generic diversity is relatively high but dominated by Acerentomidae. Another striking feature of the collected dataset is that occurrence of proturans in the Arctic regions appears to be limited to regions of Beringia that were glacier-free during the Pleistocene. Emerging patterns in species distribution and their distinct morphology also imply a relict origin and a potentially high level of endemism within the proturan fauna of that region. It is clear, however, that proturans in the Arctic regions have been studied very selectively. Hence, consideration of the importance of biogeographical history and the concept of endemism is itself strongly hampered by the limited extent of available data, which highlights the need for considerably greater survey efforts. Still, this work provides a strong baseline needed to facilitate further studies addressing diversity and biogeography of these little-known arthropods in the Arctic regions and elsewhere.

## Figures and Tables

**Table 1:** A list of all records of Protura from the Arctic regions with information on their taxonomic position, and geographical and environmental distribution. – * type locality.

Species and Their Taxonomic Position	Records from the Arctic	Environmental Settings	Distribution	References
**Order Eosentomata**				
**Family Eosentomidae** Berlese, 1909				
**Genus *Eosentomon*** Berlese, 1908				
*Eosentomon adakense* Bernard, 1985	Kuluk Bay *, Adak Island, Aleutian Islands (Bering Sea), Alaska, USA (51°50′ N, 176°40′ W).	Soil, beach terrace with dune grass (*Elymus mollis*).	Alaska (type area only).	[14]
*Eosentomon alaskaense* Nosek, 1977	Finger Mountain (671 m elev.), Yukon-Koyukuk, Alaska, USA (66°21′30″ N, 150°27′30″ W). Mastodon Dome * at Eagle Creek, Crazy Mountains, Yukon-Koyukuk, Alaska, USA (65°26′ N, 145°21′ W). Nunivak Island (Bering Sea), Alaska, USA (60°06′ N, 166°30′ W).	* 1200 m elev., on exposed cliff in *Dryas* sp.	Alaska.	[11]
*Eosentomon aquilinum* Nosek, 1980	Denali National Park and Preserve * (as Mt. McKinley N.P.) between Teklanika River and Sanctuary River, Alaska, USA (63°40′ N, 149°30′ W).	Soil, relatively dry slope with dead aspen.	Alaska (type area only).	[12]
*Eosentomon copelandi* Nosek, 1980	Denali National Park and Preserve * (as Mt. McKinley N.P.), Eielson, Alaska, USA (63°25′ N, 150°20′ W).	Soil, alpine tundra below talus, grass tussocks and moss.	Alaska (type area only).	[12]
*Eosentomon* sp. (“larva 2”, indeterminable to species)	Reindeer Station, Northwest Territories, northern Canada (68°42′ N, 134°08′ W).	Moist soil with lichens.	–	[10]
**Order Acerentomata**				
**Family Acerentomidae** Silvestri, 1907				
*Acerentulus* sp. (undetermined species).	Umiat Mt., Alaska, USA (69°23′30″ N, 152°01′18″ W).	Humus, below the summit, above the Colville River at elevation 900 feet (ca.275 m), the warmest exposure on the south side of the bluff.	–	[23]
*Acerentulus* sp. (undetermined species).	Anaktuvuk Pass, Alaska, USA (68°10′ N, 151°45′ W).	Humus, about lichens, mosses, and higher plant roots, at elevation 3950 feet (ca.1200 m) on fully exposed jagged rocks facing the pass.	–	[23]
**Subfamily Acerentominae** Silvestri, 1907				
**Genus *Fjellbergella*** Nosek, 1978				
*Fjellbergella tuxeni* Nosek, 1980	Chena Ridge, Fairbanks, Alaska, USA (64°50′ N, 147°57′ W). Brushkana River E of Cantwell *, Denali, Alaska, USA (63°20′ N, 148°10′ W). Denali National Park and Preserve (as Mt. McKinley N.P.), Denali, Alaska, USA (63°00′ N, 151°00′ W).	*Soil, tundra with moss, lichens, grass, *Ledum* sp., *Vaccinium* sp., thick turf.	Alaska.	[12]
**Genus *Orinentomon*** Yin, Xie, 1993				
*Orinentomon greenbergi* (Nosek, 1980) syn. *Yamatentomon greenbergi* Nosek, 1980	Denali National Park and Preserve * (as Mt. McKinley N.P.), Eielson, Alaska, USA (63°25′ N, 150°20′ W).	Soil; moss, grass, *Salix* in alpine tundra talus.	Alaska (type area only).	[12]
**Genus *Sugaentulus*** Imadaté, 1978				
*Sugaentulus hoogstraali* (Nosek, 1980) syn. *Nosekiella hoogstraali* Nosek, 1980	Smith Lake *, Fairbanks, Alaska, USA (64°52′ N, 147°52′ W). Denali National Park and Preserve (as Mt. McKinley N.P.), Eielson, Alaska, USA (63°25′ N, 150°20′ W).	* Soil and litter, deep rotten layer in squirrel mound of white spruce cones. Soil, moss, grass, *Salix* in alpine tundra below talus.	Alaska.	[12,20]
*Sugaentulus andrzeji* Shrubovych & Rusek, 2010	Turukhansk area *, Evenkia, Krasnoyarskiy Kray, Siberia, Russia (65°48′ N, 88°00′ E).	Soil, litter, and moss in birch, mixed and montane coniferous forests with dense herbaceous cover.	Northern* and Southern Siberia.	[18,26]
**Genus *Tuxenentulus*** Imadaté, 1973				
*Tuxenentulus boedvarssoni* Nosek, 1981	Chena Ridge *, Fairbanks, Alaska, USA (64°50′ N, 147°57′ W).	Humus and dry litter in a mixed alder, aspen, birch forest.	Alaska (type area only).	[13]
**Genus *Yamatentomon*** Imadaté, 1964				
*Yamatentomon yamato* (Imadaté & Yosii, 1956) syn. *Acerentulus yamato* Imadaté & Yosii, 1956 *Acerentomon yamato*, *Acerella yamato, Yamatentomon tokapchupi* Imadaté, 1964	Lena Delta Nature Reserve, Tyallakh, Sakha (Yakutia), Siberia, Russia (72°19′36″ N, 128°07′27″ E).	Soil and moss in sedge/moss tundra.	Japan *, Korea, Northern China, Russian Far East, and Northern Siberia.	[3,17,31] Current study.
**Subfamily Nipponentominae** Yin, 1983				
**Genus *Alaskaentomon*** Nosek, 1977				
*Alaskaentomon condei* Nosek, 1981	Chena Ridge *, Fairbanks, Alaska, USA (64°50′ N, 147°57′ W).	Dry alder litter in a mixed alder-aspen-birch forest.	Alaska (type area only).	[13,21]
*Alaskaentomon fjellbergi* Nosek, 1977	Sagwon Upland *, CRREL 12, Alaska, USA (69°30′ N, 148°30′ W).	Dry exposed hill with *Dryas* sp. and *Carex rupestris*.	Alaska (type area only).	[11,21]
**Genus *Imadateiella*** Rusek, 1974				
*Imadateiella mixta* (Nosek, 1981) syn. *Verrucoentomon mixtum* Nosek, 1981	Chena Ridge *, Fairbanks, Alaska, USA (64°50′ N, 147°57′ W).	Litter in shrubs with *Betula* sp. and *Equisetum* sp., N slope, rather moist.	Alaska (type area only).	[13,22]
*Imadateiella sharovi* (Martynova, 1977) syn. *Acerella sharovi* Martynova, 1977	Turukhansk area, Evenkia, Krasnoyarskiy Kray, Siberia, Russia (65°48′ N, 88°00′ E). Aborigen Station, Kolyma River, Magadan District, Russia (61°59′ N, 149°20′ E). Snow Valley *, Magadan area, Magadan District, Russia (59°40′ N, 150°25′ E).	Soil, litter, and turf in dry localities, under lichens and shrubs.	Northern Siberia and Russian Far East.	[15,18]
**Genus *Nienna*** Szeptycki, 1988				
*Nienna chukotka* Shrubovych, 2019	Akanotenmeem hill *, near Apapelgino village, Chaunskiy district, Chukotka, Russia (69°48′40″ N, 170°35′51″ E).	Soil in dry locality with *Dryas* sp., 20 m elev.	Northern Siberia (type area only).	[27]
**Genus *Mastodonentomon*** n. gen.				
*Mastodonentomon macleani* (Nosek, 1977) syn. *Nipponentomon macleani* Nosek, 1977	Mastodon Dome * at Eagle Creek, Crazy Mountains, Yukon-Koyukuk, Alaska, USA (65°26′ N, 145°21′ W).	1200 m elev., on exposed cliff in *Dryas* sp.	Alaska (type area only).	[11]
**Genus *Vesiculentomon*** Rusek, 1974				
*Vesiculentomon condei* (Tuxen, 1955) syn. *Acerentulus condei* Tuxen, 1955; *Acerella condei*; *Nosekiella condei*.	Richardson Mountains *, Yukon, northern Canada (68°24′ N, 135°37′ W).	600 m elev., dry locality with *Dryas* sp.	Northern Canada (type area only).	[10,20]
**Genus *Nosekientomon*** Shrubovych, Rusek, Bernard 2014				
*Nosekientomon ruseki* (Nosek, 1977) syn. *Vesiculentomon ruseki* Nosek, 1977	Mastodon Dome * at Eagle Creek, Crazy Mountains, Yukon-Koyukuk, Alaska, USA (65°26′ N, 145°21′ W).	1200 m elev., on exposed cliff in *Dryas* sp.	Alaska (type area only).	[11,19]
**Genus *Verrucoentomon*** Rusek, 1974				
*Verrucoentomon canadense* (Tuxen, 1955) syn. *Acerentulus canadensis* Tuxen, 1955, *Acerella canadensis; Nosekiella canadensis; Verrucoentomon canadensis.*	Reindeer Station, Northwest Territories, northern Canada (68°42′ N, 134°08′ W). Richardson Mountains *, Yukon, northern Canada, (68°24′ N, 135°37′ W).	130 m elev., moist localities with moss, Cladinia, Empetrum, cowberries and *Betula nana*. *600 m elev., dry locality with *Dryas* sp.	Northern Canada.	[10,22]
*Verrucoentomon imadatei* Nosek, 1977 syn. *Acerentulus imadatei* Nosek, 1977	Meade River at Atqasuk *, North Slope, Alaska, USA (70°29′ N, 157°25′ W).	Litter and humus in *Salix*-shrub on sandy river slope.	Alaska (type area only).	[11,22]
*Verrucoentomon anatoli* Shrubovych & Bernard, 2012	Turukhansk area *, Evenkia, Krasnoyarskiy Kray, Siberia, Russia (65°48′ N, 88°00′ E). Mirnoye area, Turukhanskiy District, Krasnoyarskiy Kray, Siberia, Russia (62°35′ N, 89°03′ E). Podkamennaya Tunguska River (bank), Borskiy District, Krasnoyarskiy Kray, Siberia, Russia (61°70′ N, 90°63′ E).	Soil, moss, and litter in mixed taiga forests.	Northern and southern Siberia.	[16,18]
**Genus *Yavanna*** Szeptycki, 1988				
*Yavanna babenkoi* Shrubovych, Rusek & Bernard 2012	Turukhansk area *, Evenkia, Krasnoyarskiy Kray, Siberia, Russia (65°48′ N, 88°00′ E). Mirnoye area, Turukhanskiy District, Krasnoyarskiy Kray, Siberia, Russia (62°35′ N, 89°03′ E).	Soil, litter, and moss in mixed and taiga forest.	Northern and southern Siberia.	[17,18]
*Yavanna behanae* (Nosek, 1977) syn *Nosekiella behanae* Nosek, 1977	Gobbler Knobb * (920 m elev.), Yukon-Koyukuk, Alaska, USA (67°28′30″ N, 150°11′30″ W).	Moist lichen slope in paper birch stand.	Alaska (type area only).	[11]

**Table 2:** Body chaetotaxy of *Mastodonentomon macleani* (Nosek, 1977).

	Dorsal		Ventral	
Segment	Formula	Setal Composition	Formula	Setal Composition
Th. I	4	1, 2	4 + 4 6	A1, 2, M1, 2 P1, 2, 3
Th. II	8 + 2 16	A1, 2, 3, 4, M P1, 1a, 2, 2a, 3, 3a, 4, 5	5 + 2 4	Ac, 2, 3, M P1, 3
Th. III	8 + 6 16	A1, 2, 3, 4, M1, 2, 3 P1, 1a, 2, 2a, 3, 3a, 4, 5	9 + 2 4	Ac, 1, 2, 3, 4, M P1, 3
Abd. I	8 12	A1, 1’, 2, 5 P1, 1a, 2, 2a, 3, 4	5 4	Ac, 2, 3 P1, 1a
Abd. II-III	12 16	A1, 1’, 2, 3, 4, 5 P1, 1a, 2, 2a, 3, 4, 4a, 5	5 5	Ac, 1, 2 Pc, 1a, 2
Abd. IV-VI	10 16	A1, 2, 3, 4, 5 P1, 1a, 2, 2a, 3, 4, 4a, 5	5 8	Ac, 2, 3 P1, 1a, 2, 3
Abd. VII	10 16	A1, 2, 3, 4, 5 P1, 1a, 2, 2a, 3, 4, 4a, 5	3 9	Ac, 2 Pc, 1, 1a, 2, 3
Abd. VIII	6 15	A1, 4, 5 Pc, 1, 1a, 2, 2a, 3, 3a, 5	4 2	1, 2 P1a
Abd. IX	12	1, 1a, 2, 2a, 3, 4	4	
Abd. X	10	1, 2, 2a, 3, 4	4	
Abd. XI	6	1, 3, 4	6	
Abd. XII	9		6	
